# Help-Seeking in the Age of AI: Cross-Sectional Survey of the Use and Perceptions of AI-Based Mental Health Support Among US Adults

**DOI:** 10.2196/88196

**Published:** 2026-03-30

**Authors:** Michiko Ueda, Michael L Birnbaum, Yanhong Liu, Qingyi Yu, Xihe Tian, Anna Mirer, Seethalakshmi Ramanathan, Mark Sinyor

**Affiliations:** 1 Department of Public Administration and International Affairs Maxwell School of Citizenship and Public Affairs Syracuse University Syracuse, NY United States; 2 Center for Policy Research Maxwell School of Public Affairs and Citizenship Syracuse University Syracuse, NY United States; 3 Department of Psychiatry Vagelos College of Physicians and Surgeons Columbia University New York, NY United States; 4 Behavioral Health Services and Policy Research New York State Psychiatric Institute New York, NY United States; 5 School of Education Syracuse University Syracuse, NY United States; 6 The Barnes Center at The Arch Syracuse University Syracuse, NY United States; 7 The Lerner Center for Public Health Promotion Maxwell School of Citizenship and Public Affairs Syracuse University Syracuse, NY United States; 8 Department of Psychiatry Sunnybrook Health Sciences Centre Toronto, ON Canada; 9 Department of Psychiatry University of Toronto Toronto, ON Canada

**Keywords:** help-seeking behavior, mental health, counseling, artificial intelligence, health care professionals, suicidal ideation

## Abstract

**Background:**

Anecdotal evidence suggests that an increasing number of people are turning to generative artificial intelligence (GenAI) tools or artificial intelligence (AI)-assisted chatbots to discuss and manage mental health concerns. However, systematic data on the use and perception of such tools remain scarce.

**Objective:**

This study aimed to examine how young and middle-aged adults in the United States use GenAI and AI-assisted mental health chatbots as mental health resources and assess their preferences for these tools relative to human mental health professionals.

**Methods:**

An anonymous online survey was conducted in October 2025 among US adults in a commercial online panel sample of US adults aged 18-49 years (N=1805). Respondents were asked about the sources they typically turn to when facing mental health concerns, their frequency of using GenAI tools or chatbots for mental health support, and whether the frequency of seeing human mental health professionals had changed since they started using AI tools for mental health support. Attitudes toward AI-based mental health support were assessed and compared with attitudes toward human mental health professionals.

**Results:**

In this sample, of the 1805 respondents, 638 (35.2%) reported using AI tools at least once a week in a typical week for mental health support, and 99 (5.5%) were classified as “heavy users” who reported regularly spending hours discussing their mental health concerns through AI. However, nearly 60% of respondents reported that they would turn first to family (1078/1805) and friends (1046/1805) when facing mental health concerns. Respondents who screened positive for moderate to severe depressive or anxiety symptoms were more likely to use AI-based mental health support compared to those without these symptoms (adjusted odds ratio 1.71, 95% CI 1.36-2.15) and those with suicidal ideation were more likely to be heavy AI users (adjusted odds ratio 2.42, 95% CI 1.49-3.95). Among those who had ever seen a human mental health professional (n=511), 28.4% (145/511) reported a perceived decline in visit frequency to human mental health professionals since they started using AI tools for the same purpose. Participants expressed more favorable attitudes toward human mental health professionals than toward AI-based tools. However, among heavy AI users, perceptions of AI-based mental health support and human counseling were nearly equivalent in positivity.

**Conclusions:**

AI appears to be an important component of the mental health help-seeking landscape among respondents in this sample. Although most respondents still preferred human professionals, a subset reported relying on AI tools for comparable support. Ongoing monitoring and ethical guidelines are needed to ensure that AI technologies expand access to care while being safely and effectively integrated into the broader continuum of mental health services.

## Introduction

In the last few years, generative artificial intelligence (GenAI) has rapidly entered people’s daily lives, providing information, advice, and emotional companionship. Anecdotal reports and media coverage indicate that an increasing number of people are using general-purpose GenAI tools, such as ChatGPT (OpenAI), to discuss mental health concerns [1-4], despite the fact that these systems were not specifically designed for counseling purposes. Parallel to this trend, artificial intelligence (AI)-assisted wellness apps and chatbots have gained significant traction for the purpose of improving and managing mental health [5]. Examples include Woebot (Woebot Health) and Replika (Luka), which are explicitly marketed for mental health support.

Concerns have been raised within the mental health professional community in response to these rapid and profound developments that can fundamentally change how people interact with both AI tools and human counselors. Some clinicians warn that AI-based mental health support could eventually replace human counselors [6,7]. Others have highlighted potential risks associated with AI chatbot use; for example, the so-called “AI-induced psychosis” phenomenon, in which users reportedly develop psychosis-like symptoms as a result of interactions with AI tools [8-12].

Although AI-based mental health tools are widely discussed in media and policy contexts, empirical evidence remains sparse. A recent nationally representative study of US adolescents and young adults found that 13.1% reported using GenAI for mental health advice, with markedly higher rates among those aged 18-21 years [13]. Other recent studies have focused on specific subpopulations, such as college students [14,15] or active users of companion AI [16], leaving usage patterns among the broader population largely unknown. Thus, little is known about how AI tools are used for mental health purposes among adults, how people perceive such tools relative to human mental health professionals, and whether reliance on AI tools might affect traditional help-seeking behavior.

This study addresses these gaps by investigating how young and middle-aged adults in the United States use GenAI and AI-assisted chatbots for mental health support (hereafter “AI-based mental health support”) and how they perceive such tools relative to human mental health professionals. We also examine whether the use of AI-based mental health support is associated with decreased engagement with human counseling services. By focusing on individuals aged 18-49 years, our aim is to capture the experiences and perceptions of a population of adults that is both highly digitally engaged and at elevated risk for mental health conditions compared to older adults [17-19].

## Methods

### Overview

We conducted an anonymous online survey among individuals in the United States between October 24 and 27, 2025. Respondents were recruited using a commercial online panel (CloudResearch’s Connect platform, an online participant recruitment system that provides access to a large pool of vetted respondents) among those aged 18-49 years. Study invitations described the survey as focusing on perceptions and attitudes toward mental health help-seeking and did not refer to AI. The number of questions was 14, and the order of some questions was randomized, as described below. Quota sampling was applied for sex, age group (18-29, 30-39, and 40-49 years), race, and ethnicity, based on population estimates from the American Community Survey [20]; however, because participants were recruited from a commercial panel, the sample should not be considered nationally representative. All questions required responses in order to proceed, and thus the dataset contained no missing values. The final sample size was 1805. The survey took approximately 5 minutes to complete on average.

### Sociodemographic Variables

Respondents’ sociodemographic information included gender, race, ethnicity, partnership status, household income group, educational attainment, and occupation, which was provided by the survey company, instead of being collected in the survey. For logistic regression analyses, several variables were coded as binary indicators. “No partner” was coded as 1 for those not married, partnered, or in a civil union; “No college” was coded as 1 for respondents without a college degree, or who preferred not to say; and “Not in labor force” was coded as 1 for those reporting unemployment, student status, retirement, not in paid work (eg, homemaker), or “prefer not to say.” Household income was categorized into 4 levels (<US $40k, US $40-74k, US $75-124k, and ≥US $125k) and a combined missing and prefer-not-to-say group (including both item nonresponse and intentional nondisclosure).

### Mental Health Conditions and Suicidal Ideation

Depressive and anxiety symptoms were measured using the 4-item Patient Health Questionnaire (PHQ-4) [21]. We used the standard cutoff score of 6 [22] as an indication of the presence of moderate to severe depressive or anxiety symptoms. Past-year suicidal ideation was measured by asking, “How often have you thought about killing yourself in the past year?” Respondents who answered “Sometimes,” “Often,” or “Very Often” were classified as having suicidal ideation (“Suicidal Ideation”).

### Number of Confidantes

As a measure of social connection, respondents were asked approximately how many friends they feel close to, can confide in, or talk to about personal problems. This question was adopted from an earlier work [23]. Those who selected “0” were coded as “No confidante.”

### Usage of GenAI or Chatbots and Mental Health Professionals

To measure the usage of GenAI and chatbots, respondents were asked, “In a typical week, on how many days do you use GenAI tools or chatbots to discuss or deal with your mental health concerns?” The response options ranged from “0 days” to “every day”. If they indicated any use, they were further asked about the average time spent per day on GenAI or chatbots to discuss or deal with their mental health concerns, ranging from “less than 10 minutes” to “more than 3 hours.”

We created a dichotomous variable “AI use” for respondents who answered that they typically use GenAI and chatbots as a mental health resource 1 or more days per week. We also created a binary variable “Heavy AI Use” for respondents who reported using GenAI tools or chatbots for mental health purposes either daily, on 5 to 6 days per week for 30 minutes or longer per day, or on 3 to 4 days per week for 1 hour or longer per day.

In sensitivity analyses, we also examined alternative definitions of heavy AI use to ensure that our results were not sensitive to the threshold used to define “Heavy AI Use.” Specifically, we considered (1) a frequency-only definition based on near-daily use (5-6 days per week or every day) and (2) a duration-based definition emphasizing prolonged daily engagement on multiple days per week (≥1 hour per day on ≥3-4 days per week).

Respondents were also asked whether they had “ever sought help from a mental health professional (eg, psychologist, psychiatrist, therapist, counselor).” They could select one answer from the following 3 options: “Yes, I am currently seeing one,” “Yes, I have in the past but not currently,” or “Never”. The respondents who selected either of the first 2 options are classified as “Current” and “Only past.” We also created a dichotomous variable, “Ever had MH Counseling,” for those who have ever seen a mental health professional.

Respondents who reported both seeking help from a mental health professional (either currently or in the past) and using GenAI or chatbots were also asked whether their use of human mental health professionals had changed since they began using these AI tools.

### Mental Health Resources

Respondents were asked to choose up to 3 resources that they usually turn to for support or advice when they have mental health concerns. They were told that “mental health concerns” in this study referred to issues ranging from personal difficulties to mental health conditions. We provided a list of 7 choices, and the order of the choices was randomized for each respondent to reduce order bias. In addition, they could choose “Other” and enter their own response. The option “No one/I usually handle problems on my own” was also provided as an exclusive choice. Respondents who did not choose “No one” were asked to rank the 3 sources in the order of likelihood that they would turn to for support or advice.

### Attitudes Toward AI and Human Mental Health Professionals

Attitudes toward AI-based tools and human counseling were measured using a modified version of the Mental Help-Seeking Attitudes Scale (MHSAS) [24], as in [15]. The MHSAS items were presented under two hypothetical scenarios, following the design of a recent study [15]: (1) “If I had a mental-health concern, seeking help from [source] would be…” and (2) “If I were experiencing suicidal thoughts (that is, thoughts about wanting to die or end my life), seeking help from [source] would be…”. Using these scenarios allowed us to assess whether attitudes toward AI-based versus human support differed across distinct hypothetical contexts. Respondents were randomly assigned to 1 of the 2 hypothetical scenarios, and each rated both human mental health professionals and AI-based tools or chatbots (presented as “Generative-AI tools or chatbots”) within the assigned scenario. The order of presentation (AI first or human first) was randomized to minimize potential order effects.

Each stem statement was followed by 9 adjective pairs, such as “useless-useful,” “satisfying-dissatisfying,” and “healthy-unhealthy.” Respondents rated each pair on a 7-point scale, with 0 representing a neutral position and 3 on either end representing strong agreement with the adjective. The full list of adjective pairs used in this study is provided in Table S1 in Multimedia Appendix 1. As in the original study [24], the valence of adjective pairs was counterbalanced so that positive adjectives did not always appear on the same side. All items were recoded so that higher scores consistently represented more positive attitudes, and item responses were averaged to create a composite MHSAS score for each source-scenario combination. Therefore, higher scores indicate more favorable attitudes toward seeking help from the specified source. Cronbach α values ranged from 0.92 to 0.96 across the 4 conditions (human or AI × 2 scenarios), indicating excellent internal consistency.

### Statistical Analysis

This study was primarily exploratory in nature and was not designed as a formal hypothesis-testing study. Our primary analytic focus was on two focal outcomes: (1) any use of AI tools for mental health–related purposes and (2) heavy AI use, as defined in the Methods. Multivariable regression analyses examining associations with these outcomes were conducted to characterize patterns of use rather than to test prespecified causal hypotheses.

Descriptive analyses were first performed to summarize respondents’ use of GenAI tools by sociodemographic characteristics, mental health conditions, and use of mental health professionals.

To examine sociodemographic and mental health–related factors associated with the use of AI tools, logistic regression models were estimated with two binary dependent variables: (1) AI use, indicating their use of GenAI tools or chatbots for mental health support in a typical week, and (2) Heavy AI use, indicating frequent and intensive use as defined in the previous section. Independent variables included gender, age group, race and ethnicity, partnership status, income group, educational attainment, employment status, presence of confidantes, depressive and anxiety symptoms (PHQ-4≥6), and suicidal ideation. Adjusted odds ratios (aORs) and 95% CIs were reported.

As a robustness check, we conducted post hoc data-quality assessments based on implausibly short survey completion time (<1 minute) and extreme invariant responding on the MHSAS adjective-pair items using raw (ie, non–reverse-coded) responses. The median completion time was 222 (IQR 163-326) seconds (N=1805), and 5 respondents (0.28%) completed the survey in less than 1 minute. Extreme invariant responding, defined as selecting the identical response option across all 9 adjective pairs for both MHSAS item blocks within the respondent’s assigned scenario, was observed for 11 respondents (0.61%). Only 1 respondent (0.06%) met both criteria (completion time <1 minute and extreme invariant responding); excluding this conservatively flagged case would yield an analytic sample of 1804.

In addition to these respondent-level checks, we considered potential panel-level threats to response validity. The survey was administered through CloudResearch’s Connect platform, which incorporates panel-level protections such as account verification procedures and restrictions on duplicate participation. Because the questionnaire consisted entirely of closed-ended behavioral and attitudinal items and did not include open-ended responses, AI-generated text detection methods were not applicable. The instrument required respondents to report personal experiences across multiple related items (eg, mental health history, counseling use, and frequency of AI use), which reduces the plausibility of automated or scripted response patterns. No additional bot- or AI-detection tools were applied beyond these procedures.

We also conducted descriptive analyses examining self-reported changes in contacts with human mental health professionals following AI use (seeing professionals more, less, or about the same), overall and by key respondent characteristics.

To compare attitudinal differences, mean MHSAS scores were calculated for each source of help under the 2 scenarios. We compared attitudes toward human versus AI-based support within each scenario to assess whether participants evaluated AI-based mental health support differently from human counseling using paired-samples *t* tests. We report mean differences with 95% CIs and standardized effect sizes (Cohen *d*) in addition to *P* values. We then compared attitudes toward human counseling across the 2 scenarios, as well as attitudes toward AI-based mental health support across the 2 scenarios. Because only half of the respondents were presented with each scenario, the latter 2 comparisons were conducted using Welch *t* tests for independent samples.

Next, we conducted subgroup analyses to explore whether these attitudes varied by respondent characteristics. Four binary subgroup variables were examined: AI Use, Heavy AI Use, Suicidal Ideation, and Ever had MH Counseling. Between-group differences (eg, AI users vs non-AI users) were assessed using Welch *t* tests. We also conducted within-group comparisons, in which we used paired-samples *t* tests to examine whether the same set of respondents rated AI-based mental health support differently from human counseling. The main purpose of the subgroup analyses was to examine both between- and within-subgroup differences; therefore, we did not test whether attitudes differed across the 2 scenarios (mental health concerns vs suicidal thoughts). In all tests, Bonferroni corrections were applied within each family of comparisons to adjust for multiple testing.

All analyses were conducted using Python (version 3.8; Python Software Foundation). All statistical tests were 2-tailed, and a *P* value <.01 was considered statistically significant. Analyses were unweighted because the purpose of this study was to conduct exploratory between-group tests rather than to produce population-level prevalence estimates. Accordingly, reported proportions and associations are intended to characterize patterns within this sample and should not be interpreted as nationally representative estimates.

### Ethical Considerations

The Institutional Review Board of Syracuse University reviewed the study protocol, determined that it met institutional ethical standards, and deemed it to be exempt from federal regulations (#25-359). Respondents were informed about the purpose and approximate duration of the survey at the beginning and were notified that the survey contained sensitive content. They were also told that the survey would be anonymous and that no personally identifiable information would be collected. Informed consent was obtained from all participants at the beginning of the survey. They could leave the survey at any time by closing the browser. The study did not include real-time risk assessment or embedded crisis resources. Respondents were compensated for their time and effort.

## Results

### Sample Characteristics

The sample characteristics are provided in Table 1. About half were women (904/1805, 50.1%), and a majority were Non-Hispanic White (1029/1805, 57%). Age was evenly distributed, with roughly one-third in each of the age groups (18-30, 30-39, and 40-49 years). Nearly half (886/1805, 49.1%) reported having no partner. Most respondents fell into the low- or middle-income range, and just over one-third (694/1805, 38.4%) had not completed a college degree. About one-quarter (486/1805, 26.9%) were not in paid work, and 14.8% (268/1805) reported having no close friends or confidants. Approximately one-third (621/1805, 34.4%) had a score of 6 or higher for PHQ-4, suggesting moderate to severe depressive or anxiety symptoms, and 18.2% (328/1805) reported suicidal ideation during the past year. Regarding mental health counseling, 22% (398/1805) were currently seeing a professional, 48.1% (869/1805) had received counseling only in the past, and 29.8% (538/1805) had never received counseling.

**Table 1:** Descriptive statistics (N=1805).

Characteristics			Values, n (%)
**Gender**			
	Woman	904 (50.1)	
	Man	901 (49.9)	
**Age group (y)**			
	18-29	658 (36.5)	
	30-39	605 (33.5)	
	40-49	542 (30)	
**Race and ethnicity**			
	Non-Hispanic White	1029 (57)	
	Non-Hispanic Black	244 (13.5)	
	Non-Hispanic Other	178 (9.9)	
	Hispanic	354 (19.6)	
**Partner status**			
	No partner	886 (49.1)	
	Has partner	919 (50.9)	
**Income group (in US $)**			
	<40k	446 (24.7)	
	40k-74k	581 (32.2)	
	75k-124k	385 (21.3)	
	125k+	267 (14.8)	
	Missing	126 (7)	
**College degree**			
	No college degree	694 (38.4)	
	With a college degree	1111 (61.6)	
**Employment status**			
	Not in the labor force	486 (26.9)	
	In the labor force	1319 (73.1)	
**Presence of a confidante**			
	No confidante	268 (14.8)	
	Has confidantes	1537 (85.2)	
**Presence of depressive or anxiety symptoms**			
	Yes	621 (34.4)	
	No	1184 (65.6)	
**Presence of suicidal ideation**			
	Yes	328 (18.2)	
	No	1477 (81.8)	
**History of MH^a^** **counseling**			
	Current	398 (22)	
	Only past	869 (48.1)	
	Never	538 (29.8)	

^a^MH: mental health.

### Usage of GenAI or Chatbots

Table 2 reports the results of logistic regression analyses. Table S2 in Multimedia Appendix 1 reports the prevalence of AI use and heavy AI use by respondent characteristics. Among all respondents, 638 (35.3%) reported using GenAI tools or chatbots at least 1 day in a typical week to discuss or manage mental-health concerns, and 99 (5.5%) met the criteria for heavy AI use.

Non-Hispanic Black and Hispanic respondents were more likely than Non-Hispanic White respondents to use AI tools for mental health support, and Non-Hispanic Black respondents were also more likely to be heavy users. These associations remained significant after adjusting for other covariates. For example, the aORs for Non-Hispanic Black respondents were 2.094 (95% CI 1.553-2.816) for AI use and 2.773 (95% CI 1.618-4.742) for heavy AI use. Compared with those aged 18-29 years, respondents aged 30-39 years were more likely to use AI for mental health purposes (aOR 1.418, 95% CI 1.110-1.808).

Respondents with moderate to severe depressive or anxiety symptoms (PHQ-4 ≥ 6) had higher odds of using AI tools for mental health support compared with those below the threshold (aOR 1.71, 95% CI 1.359-2.151). Individuals with a history of mental health counseling were also more likely to use AI for mental health support compared with those who had never seen a human counselor. In contrast, suicidal ideation was not associated with overall AI use but was linked to higher odds of heavy AI use (aOR 2.423, 95% CI 1.487-3.947). No other consistent patterns were observed by gender, education, income, partnership status, or employment.

Across alternative definitions of Heavy AI Use, prevalence estimates were of similar magnitude, and key associations were directionally consistent (Table S5 in Multimedia Appendix 1).

The frequency of AI tools for mental health support among all respondents is shown in Figures S1 and S2 in Multimedia Appendix 1. Among AI users (n=638), a majority of them (n=428, 67%) reported using AI tools 1-2 days per week, while 53 respondents (8.3%) indicated daily use. Regarding time spent per day, 297 users (46.6% of AI users) reported spending 10-30 minutes, followed by 168 users (26.3% of AI users) who spent less than 10 minutes. A smaller subset (n=64) reported using AI tools for more than 1 hour per day.

Table 3 examines how the use of AI tools relates to subsequent reported engagement with human mental health professionals, documenting whether respondents report seeing professionals more, less, or about the same after using AI tools for similar purposes. Among AI users who had ever seen a human mental health professional (n=511), 28.4% (n=145) reported seeking services from human mental health professionals less often since starting to use AI-based tools. Among AI users who had previously received counseling but were not currently in treatment, 34.8% (118/339) reported perceived decreased visits, which is more than twice the proportion observed among those currently in counseling (27/172, 15.7%). Among current counseling users who also use AI tools, 25.6% (44/172) indicated that they see human professionals more often. Patterns also varied by AI-use intensity. Nearly half of heavy AI users (42/83, 51%) reported seeing human counselors less frequently, compared with 24.1% (103/428) among casual AI users.

**Table 2:** Artificial intelligence use and heavy artificial intelligence use by respondent characteristics: the results of logistic regressions.

Characteristics			AI^a,b^ use, aOR^c^ (95% CI)		Heavy AI use^d^, aOR (95% CI)
**Gender**					
	Woman	1.022 (0.83-1.258)		0.995 (0.651-1.52)	
	Man	Ref^e^		Ref	
**Age group (years)**					
	18-29	Ref		Ref	
	30-39	1.416 (1.107-1.81)		1.66 (0.971-2.838)	
	40-49	0.941 (0.723-1.225)		1.942 (1.114-3.384)	
**Race and ethnicity**					
	Non-Hispanic White	Ref		Ref	
	Non-Hispanic Black	2.087 (1.545-2.82)		2.771 (1.618-4.744)	
	Non-Hispanic Other	1.265 (0.881-1.817)		1.52 (0.73-3.168)	
	Hispanic	1.398 (1.075-1.817)		1.228 (0.693-2.176)	
**Partner status**					
	No partner	1.179 (0.951-1.462)		0.961 (0.617-1.498)	
	Has partner	Ref		Ref	
**Income group (in US $)**					
	<40k	1.008 (0.701-1.45)		0.728 (0.341-1.553)	
	40k-74k	1.239 (0.894-1.716)		0.801 (0.411-1.562)	
	75k-124k	0.964 (0.681-1.365)		1.109 (0.557-2.209)	
	125k+	Ref		Ref	
	Missing	1.203 (0.743-1.948)		0.809 (0.299-2.186)	
**College degree**					
	No college degree	1.159 (0.922-1.457)		1.187 (0.74-1.903)	
	With a college degree	Ref		Ref	
**Employment status**					
	Not in the labor force	0.498 (0.384-0.645)		0.45 (0.242-0.838)	
	In the labor force	Ref		Ref	
**Presence of a confidante**					
	No confidante	0.795 (0.589-1.072)		1.12 (0.639-1.965)	
	Has confidantes	Ref		Ref	
**Presence of depressive or anxiety symptoms**					
	Yes	1.71 (1.359-2.151)		1.578 (0.989-2.518)	
	No	Ref		Ref	
**Presence of suicidal ideation**					
	Yes	1.031 (0.781-1.36)		2.423 (1.487-3.947)	
	No	Ref		Ref	
**History of MH^f^** **counseling**					
	Current	2.14 (1.585-2.889)		1.851 (0.949-3.61)	
	Only past	1.908 (1.48-2.459)		1.979 (1.099-3.565)	
	Never	Ref		Ref	

^a^AI use is a dichotomous variable for those who use generative artificial intelligence and chatbots as a mental health resource one or more days per week.

^b^AI: artificial intelligence.

^c^aOR: adjusted odds ratio.

^d^Heavy AI use is defined for respondents who use AI tools for mental health purposes either daily, on 5 to 6 days per week for 30 minutes or longer per day, or on 3 to 4 days per week for 1 hour or longer per day.

^e^Ref: reference.

^f^MH: mental health.

**Table 3:** Reported changes in the frequency of seeing a mental health professional after beginning to use artificial intelligence (AI) tools.

Characteristics^a^			Total, N		Less, n (%)		Same, n (%)		More, n (%)
All			511		145 (28.4)		293 (57.3)		73 (14.3)
**Gender**									
	Woman	272		83 (30.5)		160 (58.8)		29 (10.7)	
	Man	239		62 (25.9)		133 (55.6)		44 (18.4)	
**Age groups (y)**									
	18-29	162		43 (26.5)		92 (56.8)		27 (16.7)	
	30-39	203		55 (27.1)		114 (56.2)		34 (16.7)	
	40-49	146		47 (32.2)		87 (59.6)		12 (8.2)	
**Race and ethnicity**									
	Non-Hispanic White	276		76 (27.5)		160 (58)		40 (14.5)	
	Non-Hispanic Black	90		28 (31.1)		45 (50)		17 (18.9)	
	Non-Hispanic Other	38		7 (18.4)		24 (63.2)		7 (18.4)	
	Hispanic	107		34 (31.8)		64 (59.8)		9 (8.4)	
**Partner status**									
	No partner	264		66 (25)		154 (58.3)		44 (16.7)	
	Has partner	247		79 (32)		139 (56.3)		29 (11.7)	
**Income group (in US $)**									
	<40k	115		29 (25.2)		68 (59.1)		18 (15.7)	
	40k-74k	190		56 (29.5)		107 (56.3)		27 (14.2)	
	75k-124k	95		32 (33.7)		51 (53.7)		12 (12.6)	
	125k+	76		17 (22.4)		45 (59.2)		14 (18.4)	
	Missing	35		11 (31.4)		22 (62.9)		2 (5.7)	
**College degree**									
	No college degree	198		64 (32.3)		111 (56.1)		23 (11.6)	
	With a college degree	313		81 (25.9)		182 (58.1)		50 (16)	
**Employment status**									
	Not in the labor force	94		22 (23.4)		64 (68.1)		8 (8.5)	
	In the labor force	417		123 (29.5)		229 (54.9)		65 (15.6)	
**Presence of a confidante**									
	No confidante	66		24 (36.4)		39 (59.1)		3 (4.5)	
	Has confidantes	445		121 (27.2)		254 (57.1)		70 (15.7)	
**Presence of depressive or anxiety symptoms**									
	Yes	242		63 (26)		139 (57.4)		40 (16.5)	
	No	269		82 (30.5)		154 (57.2)		33 (12.3)	
**Presence of suicidal ideation**									
	Yes	120		36 (30)		65 (54.2)		19 (15.8)	
	No	391		109 (27.9)		228 (58.3)		54 (13.8)	
**History of MH^b^** **counseling**									
	Current	172		27 (15.7)		101 (58.7)		44 (25.6)	
	Only past	339		118 (34.8)		192 (56.6)		29 (8.6)	
**Heavy AI user**									
	Yes	83		42 (50.6)		31 (37.3)		10 (12)	
	No	428		103 (24.1)		262 (61.2)		63 (14.7)	

^a^Respondents who had ever received mental health counseling and used generative artificial intelligence tools for mental health support (n=511) were asked whether their frequency of seeing a human mental health professional had changed since using AI-based tools (less, same, or more). Counts and row percentages are shown. Percentages sum to 100 within each subgroup.

^b^MH: mental health.

### Mental Health Resources

**Figure 1:**
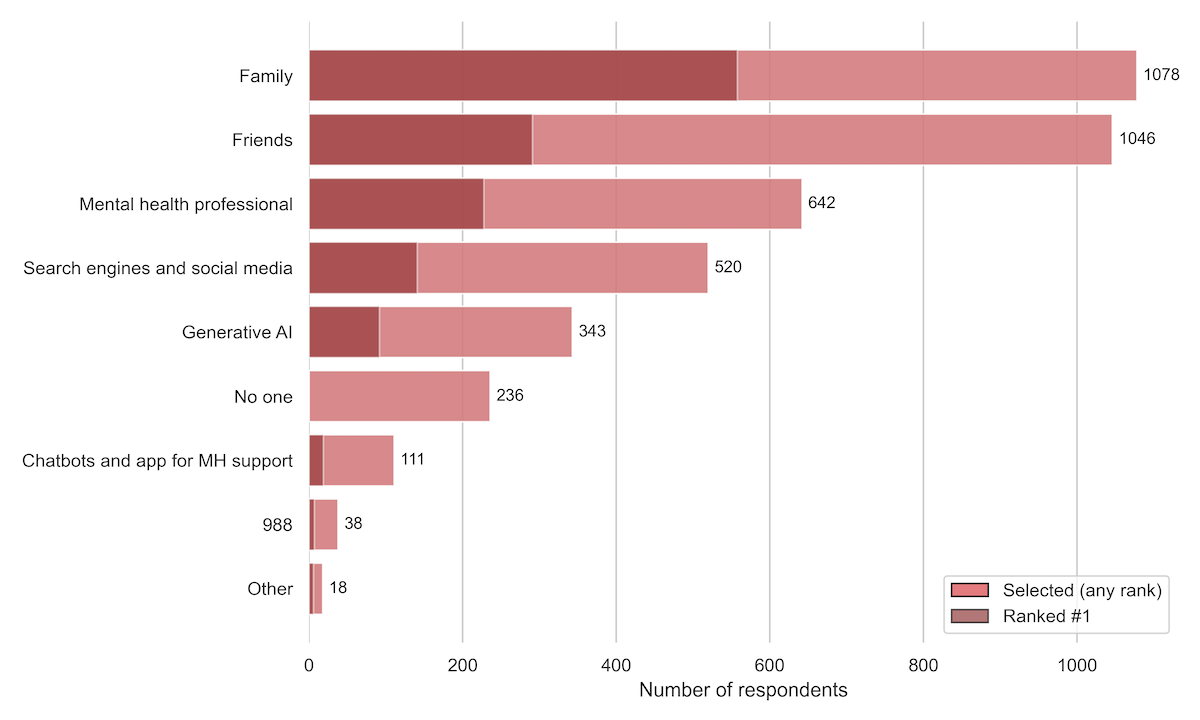
Resources that respondents can turn to for mental health support.

Moving beyond analyses restricted to AI users, Figure 1 displays the mental health resources that all respondents reported usually turning to for support or advice when they have mental health concerns. The darker portions of each bar indicate the number of respondents who selected that resource as their first choice. The vast majority of respondents reported that they would turn first to family or friends. The number of respondents who selected GenAI tools or chatbots for mental health support as one of their top 3 choices was 343 and 111, respectively. In total, 236 (13.1%) out of 1805 respondents indicated that they would turn to “No one” when facing mental health concerns.

### Attitudes Toward AI and Human Mental Health Professionals

**Figure 2:**
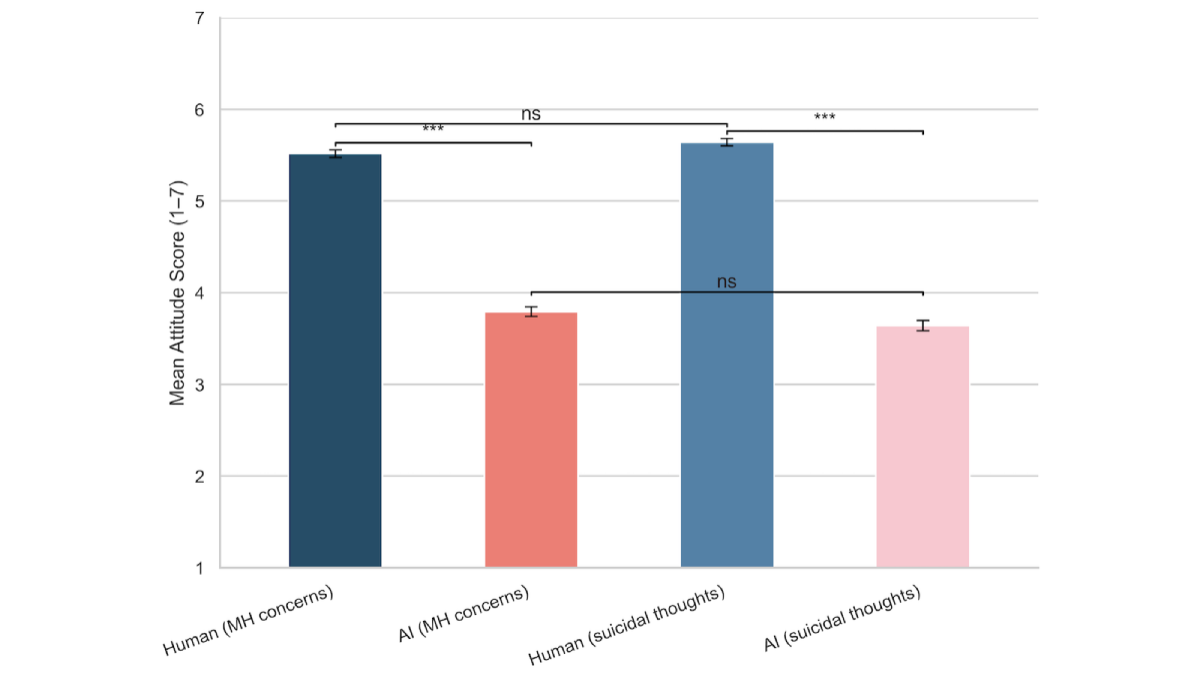
Attitudes toward seeking help from human mental health professionals versus artificial intelligence-based tools or chatbots under two hypothetical scenarios. AI: artificial intelligence; MH: mental health; ns: nonsignificant; ***: *P*<.001.

Figure 2 and Table S3 in Multimedia Appendix 1 summarize participants’ attitudes toward seeking help from human mental health professionals versus AI-based tools or chatbots, as measured by the modified MHSAS. Figures S3 and S4 in Multimedia Appendix 1 present the distributions of raw MHSAS.

Across the full sample, participants expressed significantly more favorable attitudes toward human support than AI-based support under both hypothetical scenarios. Mean MHSAS scores were lower for AI than for human professionals in the mental health concerns scenario (mean difference –1.723, 95% CI −1.847 to −1.598; Cohen *d*=1.202; *P*<.001) and in the suicidal-thoughts scenario (mean difference –2.002, 95% CI −2.130 to −1.874; Cohen *d*=1.361; *P*<.001). Attitudes toward human or AI help did not differ significantly between the 2 scenarios, suggesting that respondents evaluated the sources similarly regardless of the severity of the hypothetical condition.

When we compared respondents by their AI-use status, the presence of suicidal ideation, and the history of human mental health counseling (between-group comparisons), we found that AI users (both any and heavy users) tended to hold much more positive attitudes toward AI-based mental health support than non-AI users, while their attitudes toward human counselors did not vary by their AI-use status (Table S4 in Multimedia Appendix 1 and Figures S5 and S6 in Multimedia Appendix 1). Although there were minor differences in attitudes toward human counselors by the presence of suicidal ideation and prior counseling history, attitudes toward AI did not vary significantly by suicidality or counseling experience.

We also compared attitudes toward human versus AI-based mental health support within each subgroup (“Within-group” column in Table S4 in Multimedia Appendix 1) under the 2 hypothetical scenarios. All subgroups—except for heavy AI users under the general mental health concerns scenario—rated human mental health professionals more favorably than AI. These differences in mean scores between human and AI were statistically significant (*P*<.001) in all but one case, with AI consistently rated less positively. Heavy AI users rated AI-based support as positively as human counseling. Under the general mental health concern scenario, the mean score for human counselors among heavy AI users (n=56) was 5.294 (SD 1.593), while the corresponding mean score for AI-based support was 5.556 (SD 1.283), resulting in a statistically nonsignificant difference (difference=0.262; *P*>.99, Bonferroni-adjusted).

## Discussion

Through a survey of US adults aged 18-49 years drawn from a commercial online panel, we conducted an exploratory analysis examining how AI tools are being used for mental health support. We also assessed respondents’ attitudes toward human mental health professionals and AI-based mental health support, as well as potential perceived changes in their engagement with human counselors after beginning to use AI tools for similar purposes. Before interpreting these findings, it is important to clarify their scope. Because participants were recruited from a nonprobability panel sample, the findings should be interpreted as descriptive of this sample rather than as nationally representative estimates.

About one-third of respondents reported using AI tools more than once a week to discuss and manage mental health concerns. While most were casual users, 5.5% of all respondents were considered heavy users who reported regularly spending hours discussing their mental health concerns through AI. At the same time, the vast majority of all respondents answered that they would still turn primarily to family members and friends for mental health support, with AI tools ranking relatively low, below human counselors, as well as social media and search engines. Although human counselors were generally viewed more favorably, heavy AI users rated AI-based mental health support nearly as positively as human counseling. Roughly 1 in 4 AI users reported seeking human counseling less often since adopting AI tools, suggesting that AI use may be associated with evolving help-seeking patterns.

The overall rate of AI use for mental health purposes in this study (35.2%) differs from those reported in prior studies, which likely reflects differences in study populations, measurement, and survey timing. A survey among university students conducted in early 2024 found a 5% usage rate [15], while an early 2025 national survey of adolescents and young adults reported 13.1% [13]. Because survey wording and study populations differed across studies, these comparisons are provided for context and should not be interpreted as evidence of temporal trends.

Patterns of AI use also varied across key demographic and psychological characteristics. Some racial and ethnic minority groups had higher odds of using AI-based mental health support, and these differences remained significant after adjusting for socioeconomic and symptom-related factors. Such patterns may reflect persistent barriers to traditional care, including stigma, limited access, and cultural mistrust, which might make AI tools appealing as alternative sources of support. This aligns with the argument that AI tools may fill critical gaps in accessibility by offering low-barrier, immediate, and stigma-free mental health support, particularly in contexts where human services are scarce or unaffordable [25-27].

Beyond demographics, mental health symptom profiles also played a critical role. Those screened positive for moderate to severe depressive or anxiety symptoms (PHQ-4 ≥6) were more likely to use AI-based mental health support, likely because they had more mental health concerns to discuss. The immediate availability of AI tools might also make them attractive to individuals with sudden episodes of those conditions. At the same time, this finding may also reflect the perceived relevance or appeal of AI tools for those experiencing distress. Some studies indicate that conversational AI agents can help reduce depressive symptoms and psychological distress, including suicidal ideation [28,29], and the social need fulfillment model [30] suggests that humans may derive both concrete and symbolic benefits from responsive AI interactions, particularly during periods of loneliness or emotional vulnerability. However, we still have limited evidence on the effectiveness of AI-based mental health support, particularly in the long term and in real-world settings. Future studies should track individuals over time to understand how effectiveness evolves and how it may vary by user characteristics (eg, age) and clinical profiles.

Importantly, we also found that those with suicidal ideation were more likely to be heavy AI users, and the result was robust to alternative definitions of heavy AI use. This finding raises concern, as current GenAI systems may not yet be capable of responding appropriately to suicidal crises despite built-in safety guardrails [31,32]. Prior studies have also warned that AI interactions may be harmful for high-risk individuals [5,33,34]. These results underscore the importance of understanding usage patterns by symptom profile and evaluating how the use and risks of AI tools vary by symptom severity. At the same time, AI tools might also serve as an opportunity for early identification of at-risk individuals and as a gateway to human-based care, if deployed safely. The fact that some individuals turn to AI may therefore offer a potential intervention point for those with elevated risk, including individuals experiencing suicidal thoughts.

AI users generally rated AI-based mental health support more positively than non-users. This may help explain, in part, why some individuals reported less frequent engagement with human mental health professionals. In this study, a notable proportion (145/511, 28%) of AI users who had ever seen a human counselor reported a perceived decrease in their use of human counseling after beginning to use AI for mental health purposes. Those who had previously seen a counselor but were no longer in treatment were especially likely to report this pattern. The reasons for discontinuing professional care remain unclear, and due to the cross-sectional nature of this study, causal relationships cannot be determined. Future longitudinal work is needed to clarify whether AI use contributes to disengagement from human counseling or simply reflects changing help-seeking preferences. In addition, future studies should examine the implications of this potential substitution for mental healthcare, as the nature of the support offered by humans might differ substantially from that provided by AI. Our findings highlight the importance of additional research to determine how these tools influence users’ help-seeking behaviors over time and to evaluate their effectiveness in real-world use.

Several limitations warrant caution. First, this study was cross-sectional and based on self-reported data, which precludes causal inference. In particular, reports of changes in human counseling use reflect retrospective self-report and should be interpreted as perceived changes rather than observed behavioral shifts. Respondents’ attitudes and usage patterns may also evolve quickly over time, which cannot be captured in a single cross-sectional survey. Second, because the survey was conducted online using a commercial panel, the sample may overrepresent digitally engaged individuals, and it might not accurately represent the general population, although demographic quotas were applied for selected characteristics. In addition, respondents recruited through a commercial panel may differ from the general population in unobserved ways, including prior mental health experiences or help-seeking histories, which could influence both AI use and attitudes toward AI-based mental health support. Similarly, because this study was conducted online, the quality of some responses may be lower than in face-to-face interviews. At the same time, the online format may have mitigated social desirability bias. Moreover, although we implemented several safeguards to identify low-quality responses, we could not definitively verify that all participants completed the survey without external assistance (eg, automated tools or third-party help), which is a general limitation of anonymous online surveys. Third, the number of heavy AI users in the sample was relatively small, and thus any conclusions about this group should be interpreted with caution. More large-scale surveys are necessary to differentiate heavy AI users from casual AI users. Fourth, we did not differentiate the types of AI tools in some of our questions. In particular, when we measured attitudes toward AI tools, the term “generative-AI tools or chatbots” was used. Thus, it is possible that some respondents had general-purpose AI tools in mind, whereas others were thinking specifically about chatbots such as Replika. However, our findings regarding respondents’ preferred sources of support (Figure 1) indicate that they most likely had general-purpose GenAI in mind. Finally, because the survey was anonymous and did not include real-time monitoring or embedded crisis resources, the study did not incorporate direct support for participants who reported suicidal ideation. This reflects a common methodological tradeoff in anonymous surveys assessing suicidality, in which anonymity may facilitate candid reporting but limits the ability to intervene if risk is disclosed. Accordingly, participation may have involved some psychological burden for respondents.

Despite these limitations, this study constitutes one of the first systematic examinations of AI-based mental health support among young and middle-aged adults in the United States. The findings suggest variation in help-seeking patterns, in which AI appears to play an important role. Future research should continuously monitor the psychological and behavioral effects of AI engagement, identify groups for whom such tools may be most beneficial or harmful, clarify the conditions under which risks emerge, and determine how AI-based tools can be safely and effectively integrated into the broader continuum of mental health care for various demographic groups, including youth and older adults.

## Appendix

Multimedia Appendix 1 Additional results.

## Abbreviations

AI: artificial intelligence
aOR: adjusted odds ratio
GenAI: generative artificial intelligence
MHSAS: Mental Help-Seeking Attitudes Scale
PHQ-4: 4-item Patient Health Questionnaire

## Data Availability

Data are available from the corresponding author upon reasonable request.
