# Supplementary material for: Help-Seeking in the Age of AI: Cross-Sectional Survey of the Use and Perceptions of AI-Based Mental Health Support Among US Adults
Source: JMIR Ment Health. 2026 Mar 30;13:e88196. doi: 10.2196/88196 (PMC13077273; doi:10.2196/88196)
Supplement: Multimedia Appendix 1 [file mental_v13i1e88196_app1.pdf]

Table S1 Mental Help-Seeking Attitudes Scale (MHSAS) adjective pairs

	3	2	1	0	1	2	3	
Useless								Useful
Important								Unimportant
Unhealthy								Healthy
Ineffective								Effective
Good								Bad
Healing								Hurting
Disempowering								Empowering
Satisfying								Dissatisfying
Desirable								Undesirable

Table S2 AI use and heavy AI use by respondent characteristics

		Counts	
		**AI Use**	**Heavy AI Use**
	Total N	N (%)	N (%)
All	1805	638 (35.3)	99 (5.5)
Woman	904	332 (36.7)	53 (5.9)
Man	901	306 (34)	46 (5.1)
Age: 18-29	658	213 (32.4)	26 (4)
Age: 30-39	605	252 (41.7)	38 (6.3)
Age: 40-49	542	173 (31.9)	35 (6.5)
Non-Hispanic White	1029	327 (31.8)	46 (4.5)
Non-Hispanic Black	244	115 (47.1)	25 (10.2)
Non-Hispanic Other	178	58 (32.6)	10 (5.6)
Hispanic	354	138 (39)	18 (5.1)
No partner	886	329 (37.1)	48 (5.4)
Has partner	919	309 (33.6)	51 (5.5)
Income: <40k	446	145 (32.5)	21 (4.7)
Income: 40k–74k	581	231 (39.8)	32 (5.5)
Income: 75k–124k	385	126 (32.7)	24 (6.2)
Income: 125k+	267	90 (33.7)	15 (5.6)
Income: Missing	126	46 (36.5)	7 (5.6)
No college degree	694	244 (35.2)	37 (5.3)
With college degree	1111	394 (35.5)	62 (5.6)
Not in labor force	486	121 (24.9)	14 (2.9)
In labor force	1319	517 (39.2)	85 (6.4)
No confidante	268	83 (31)	18 (6.7)
Has confidantes	1537	555 (36.1)	81 (5.3)
With depressive/anxiety	621	274 (44.1)	50 (8.1)
No depressive/anxiety	1184	364 (30.7)	49 (4.1)
Suicidal Ideation: Yes	328	136 (41.5)	36 (11)
Suicidal Ideation: No	1477	502 (34)	63 (4.3)
MH counseling: Current	398	172 (43.2)	27 (6.8)
MH counseling: Only past	869	339 (39)	56 (6.4)
MH counseling: Never	538	127 (23.6)	16 (3)

Notes: "*AI use*" is a dichotomous variable for those who use generative AI and chatbots as a mental health resource one or more days per week. "*Heavy AI Use*" is defined for respondents who use AI tools for mental health purposes either daily, on five to six days per week for 30 minutes or longer per day, or on three to four days per week for one hour or longer per day.

Table S3 Mean Mental Help-Seeking Attitudes Scale (MHSAS) scores toward Human and AI-based mental health support under two scenarios

Mental Health Concerns (N= 901)					
(1) Human		(2) AI			
Mean	S.D.	Mean	S.D.	Diff: (2)-(1)	p-value
5.516	1.221	3.793	1.617	-1.723	0.000

Suicidal Thoughts (N= 904)					
(3) Human		(4) AI			
Mean	S.D.	Mean	S.D.	Diff: (4)-(3)	p-value
5.641	1.224	3.64	1.683	-2.002	0.000

Across Scenario Comparison			
Diff: (3)-(1)	p-value	Diff: (4)-(2)	p-value
0.126	0.116	-0.154	0.192

Notes: Mental Help-Seeking Attitudes Scale (MHSAS) scores (range = 1–7) toward human and AI-based mental-health support under two scenarios shown. Comparisons between sources and scenarios were tested with paired t-tests and Welch's t-tests for independent samples, respectively. p-values were Bonferroni-corrected for multiple comparisons.

Table S4 Attitudes toward human mental health professionals and AI tools, by selected subgroups

	N	Human Mean	Human S.D.	Human Between group (Yes - No) Diff	Human Between group (Yes - No) p-value	AI Mean	AI S.D.	AI Between group (Yes - No) Diff	AI Between group (Yes - No) p-value	Within group (AI - Human) Diff	Within group (AI - Human) p-value
**Mental Health Concerns (N=901)**											
**AI user**											
Yes	336	5.522	1.220	0.01	1.000	4.849	1.202	1.684	0.000	-0.673	0.000
No	565	5.512	1.223			3.165	1.501			-2.347	0.000
**Heavy AI user**											
Yes	56	5.294	1.593	-0.237	1.000	5.556	1.283	1.879	0.000	0.262	1.000
No	845	5.53	1.192			3.676	1.568			-1.854	0.000
**Suicidal ideation**											
Yes	165	5.249	1.361	-0.326	0.038	3.54	1.741	-0.31	0.294	-1.709	0.000
No	736	5.575	1.180			3.85	1.583			-1.726	0.000
**Ever had MH counseling**											
Yes	634	5.592	1.191	0.256	0.041	3.818	1.629	0.084	1.000	-1.774	0.000
No	267	5.335	1.274			3.734	1.588			-1.601	0.000
**Suicidal Thoughts (N=904)**											
**AI user**											
Yes	302	5.611	1.241	-0.046	1.000	4.789	1.362	1.726	0.000	-0.822	0.000
No	602	5.657	1.216			3.063	1.526			-2.594	0.000
**Heavy AI user**											
Yes	43	5.413	1.505	-0.239	1.000	5.251	1.346	1.691	0.000	-0.163	1.000
No	861	5.653	1.208			3.559	1.658			-2.094	0.000
**Suicidal ideation**											
Yes	163	5.104	1.314	-0.655	0.000	3.093	1.683	-0.667	0.000	-2.012	0.000
No	741	5.76	1.171			3.76	1.660			-2	0.000
**Ever had MH counseling**											
Yes	633	5.651	1.197	0.033	1.000	3.635	1.698	-0.014	1.000	-2.016	0.000
No	271	5.619	1.286			3.649	1.650			-1.969	0.000

Notes: Respondents rated their attitudes toward seeking help from (a) a mental health professional and (b) generative-AI tools or chatbots under one of two hypothetical scenarios: (1) when experiencing mental health concerns or (2) when having suicidal thoughts. Each respondent was randomly assigned to one scenario only. Participants evaluated nine adjective pairs on a 7-point scale. Scores were recoded so that higher values consistently indicated more favorable attitudes and then averaged across the nine items to create mean attitude scores. “Between-group” comparisons test mean differences between respondents with and without each characteristic (Yes vs. No). “Within-group” comparisons test differences between attitudes toward AI and human help sources among the same respondents.

Table S5 Heavy AI use by respondent characteristics under alternative definitions of heavy AI use: results of logistic regressions

	Heavy AI use (primary)	Heavy AI use (Alt 1)	Heavy AI use (Alt 2)
Woman	0.995 [0.651-1.52]	0.957 [0.618-1.482]	0.67 [0.377-1.191]
Man	Ref.	Ref.	Ref.
Age: 18–29	Ref.	Ref.	Ref.
Age: 30–39	1.66 [0.971-2.838]	1.393 [0.807-2.402]	1.673 [0.82-3.413]
Age: 40–49	1.942 [1.114-3.384]	1.644 [0.937-2.883]	1.58 [0.733-3.406]
Non-Hispanic White	Ref.	Ref.	Ref.
Non-Hispanic Black	2.771 [1.618-4.744]	2.757 [1.56-4.872]	2.811 [1.353-5.841]
Non-Hispanic Other	1.52 [0.73-3.168]	2.165 [1.056-4.439]	1.116 [0.369-3.381]
Hispanic	1.228 [0.693-2.176]	1.611 [0.916-2.833]	1.429 [0.68-3.003]
No partner	0.961 [0.617-1.498]	0.958 [0.609-1.509]	1.257 [0.685-2.305]
Has partner	Ref.	Ref.	Ref.
income: <40k	0.728 [0.341-1.553]	1.074 [0.458-2.517]	0.829 [0.297-2.317]
Income: 40k–74k	0.801 [0.411-1.562]	1.573 [0.747-3.314]	0.967 [0.402-2.325]
Income: 75k–124k	1.109 [0.557-2.209]	1.566 [0.716-3.425]	0.833 [0.307-2.258]
Income: 125k+	Ref.	Ref.	Ref.
Income: Missing	0.809 [0.299-2.186]	0.905 [0.286-2.861]	1.039 [0.276-3.901]
No college degree	1.187 [0.74-1.903]	1.129 [0.698-1.827]	1.087 [0.574-2.059]
With college degree	Ref.	Ref.	Ref.
Not in labor force	0.45 [0.242-0.838]	0.418 [0.217-0.807]	0.444 [0.186-1.061]
In labor force	Ref.	Ref.	Ref.
No confidante	1.12 [0.639-1.965]	1.086 [0.604-1.951]	0.955 [0.424-2.153]
Has confidantes	Ref.	Ref.	Ref.
With depressive/anxiety	1.578 [0.989-2.518]	1.506 [0.93-2.439]	2.298 [1.219-4.333]
No depressive/anxiety	Ref.	Ref.	Ref.
Suicidal ideation: Yes	2.423 [1.487-3.947]	1.98 [1.181-3.319]	2.216 [1.175-4.18]
Suicidal ideation: No	Ref.	Ref.	Ref.
MH counseling: Current	1.851 [0.949-3.61]	1.176 [0.597-2.319]	5.269 [1.727-16.071]
MH counseling: Only past	1.979 [1.099-3.565]	1.659 [0.948-2.903]	3.594 [1.226-10.537]
MH counseling: Never	Ref.	Ref.	Ref.

Notes: "*Heavy AI Use (primary)*" is defined for respondents who use AI tools for mental health purposes either daily, on five to six days per week for 30 minutes or longer per day, or on three to four days per week for one hour or longer per day. This is the definition that used in the main analysis. "*Heavy AI Use (Alt 1)*" is defined for respondents who use AI tools 5–6 days per week or every day). "*Heavy AI Use (Alt 2)*" is defined for respondents who use AI tools 1 hour or more hours per day on at least three to four days per week. The number of respondents who were classified as "*Heavy AI Use*" under each definition was 99 (primary, as reported in the manuscript), 92 (Alt 1), and 52 (Alt 2), respectively.

Figure S1 Typical frequency and duration of AI use for mental health support

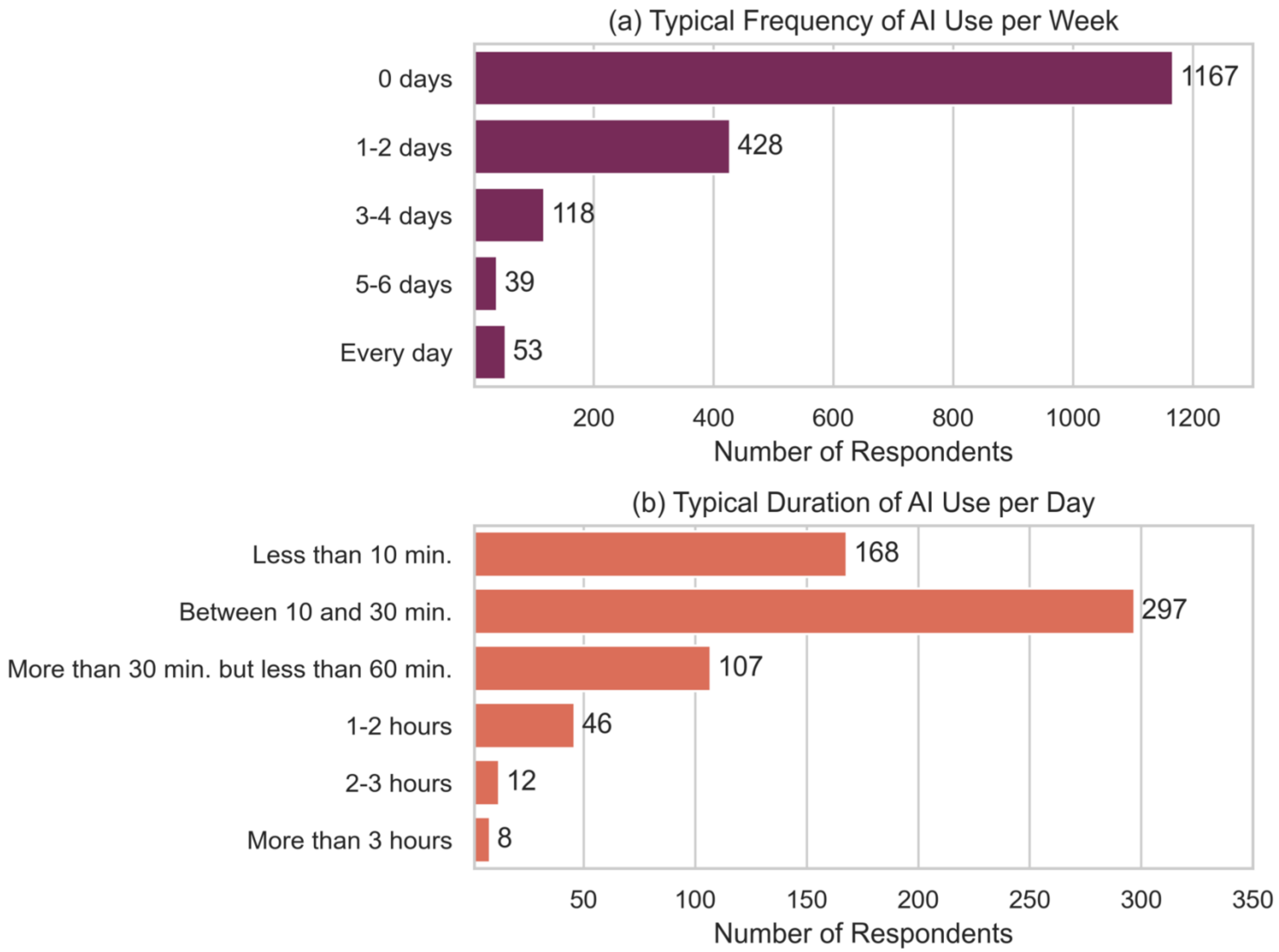

Figure S2 Frequency of generative AI-tool use for mental health support

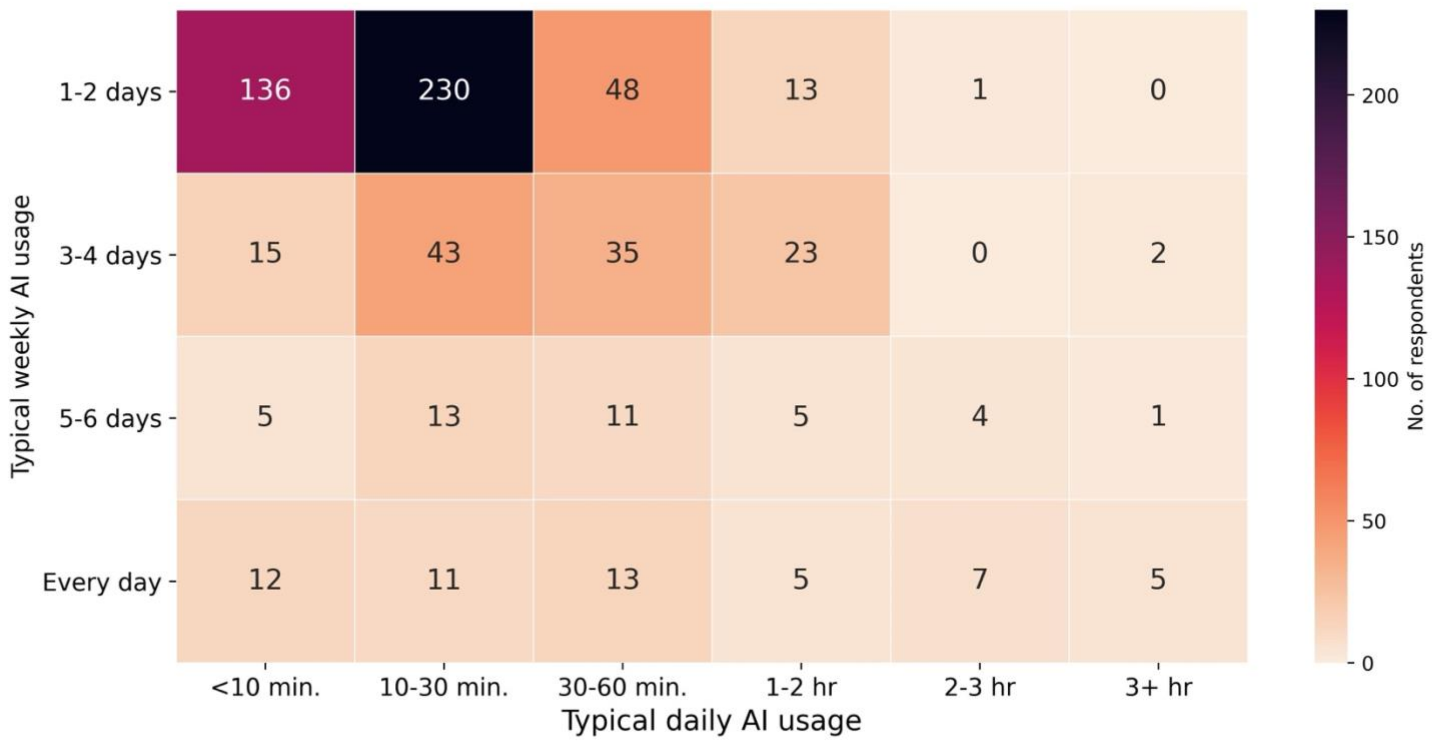

Figure S3 Distribution of Raw Mental Help-Seeking Attitudes Scale (MHSAS) Scores (Mental Health Condition Scenario)

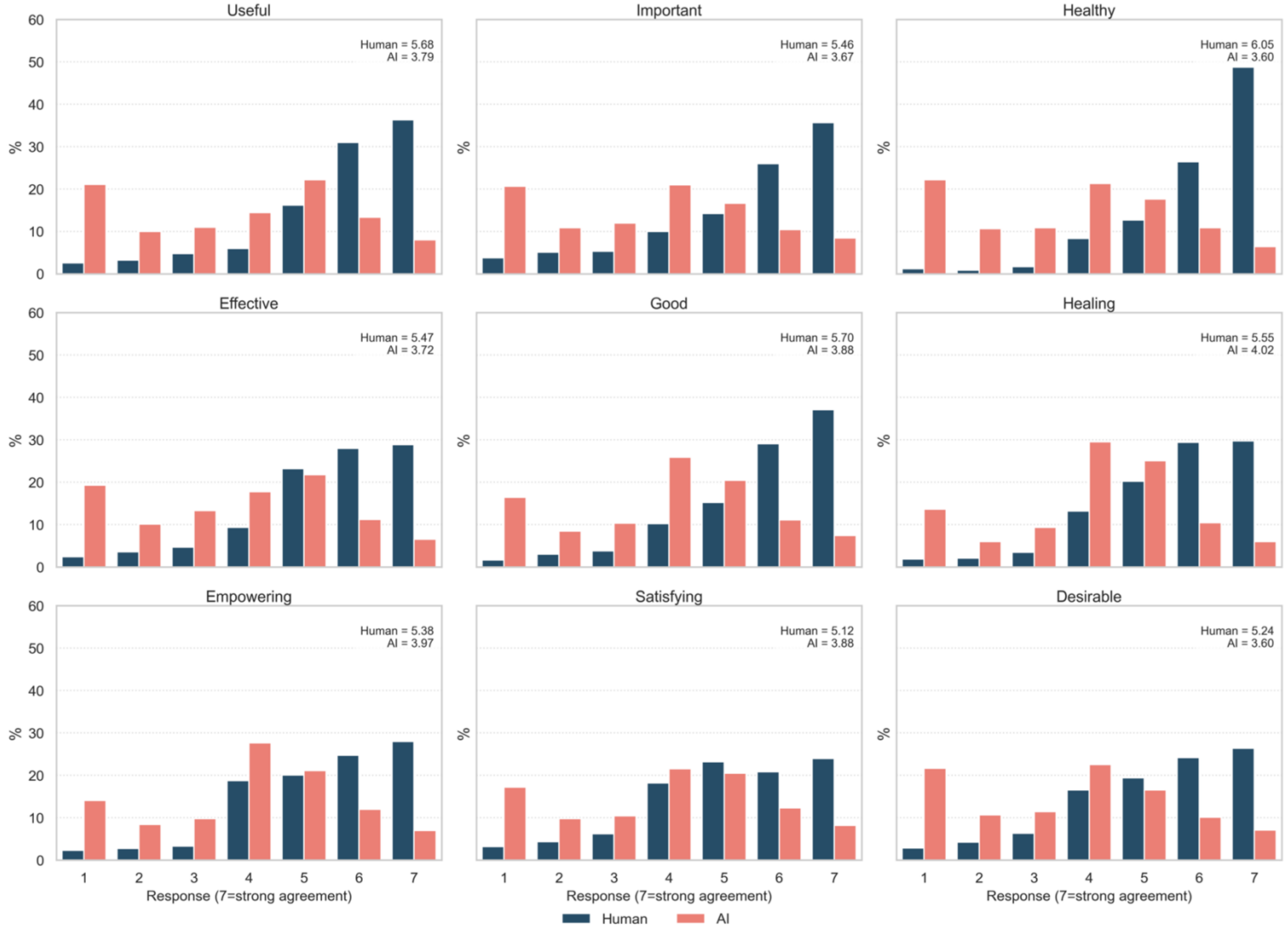

Notes: Respondents rated nine bipolar adjective pairs (e.g., “useless-useful,” “satisfying-dissatisfying”) on a 7-point scale. Items were recoded so that higher values consistently indicate more positive attitudes toward seeking help from the specified source. The figure depicts the distribution of these recoded raw scores prior to averaging them into respondent-level mean attitude scores used in subsequent analysis.

Figure S4 Distribution of Raw Mental Help-Seeking Attitudes Scale (MHSAS) Scores (Suicidal Thoughts Scenario)

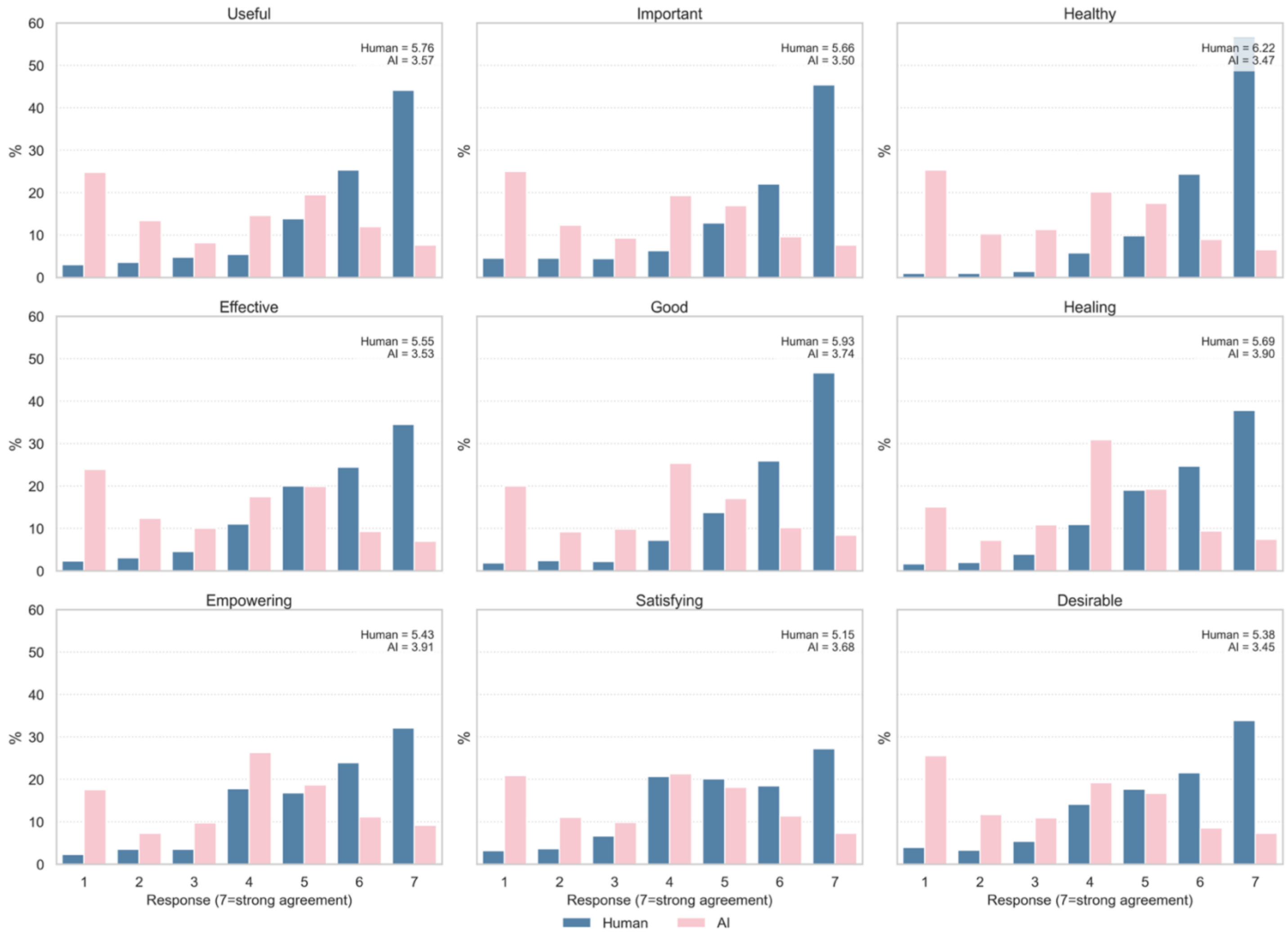

Notes: Respondents rated nine bipolar adjective pairs (e.g., "useless-useful," "satisfying-dissatisfying") on a 7-point scale. Items were recoded so that higher values consistently indicate more positive attitudes toward seeking help from the specified source. The figure depicts the distribution of these recoded raw scores prior to averaging them into respondent-level mean attitude scores used in subsequent analyses.

Figure S5 Distribution of Mental Help-Seeking Attitudes Scale (MHSAS) Scores by Selected Subgroups (Mental Health Condition Scenario)

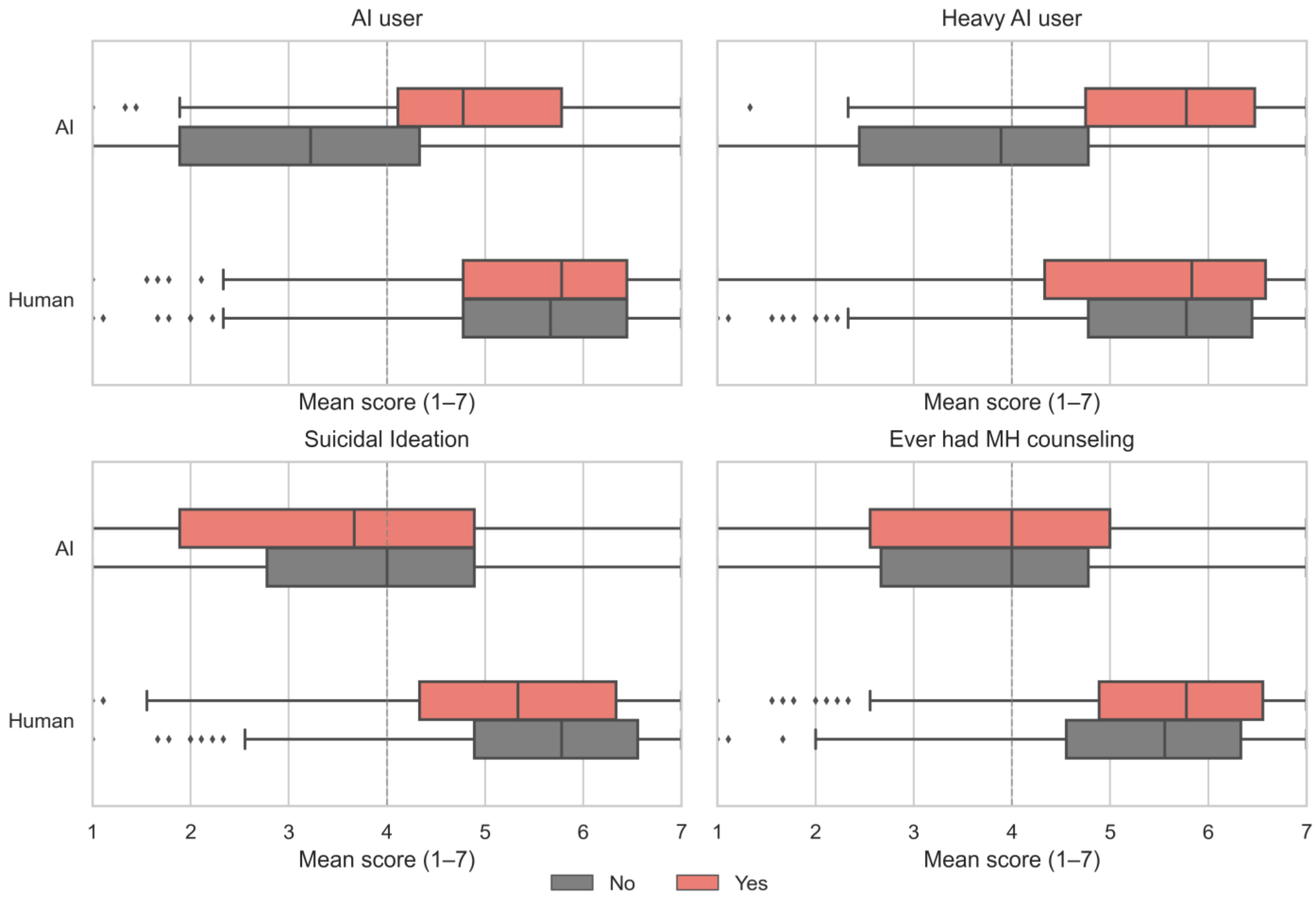

Notes: Each box represents the interquartile range (IQR; 25th–75th percentiles). The horizontal line inside the box indicates the median, and the whiskers extend to 1.5 × IQR. Circles represent individual data points outside that range. Higher scores indicate more favorable attitudes toward the specified source.

Figure S6 Distribution of Mental Help-Seeking Attitudes Scale (MHSAS) Scores by Selected Subgroups (Suicidal Thoughts Scenario)

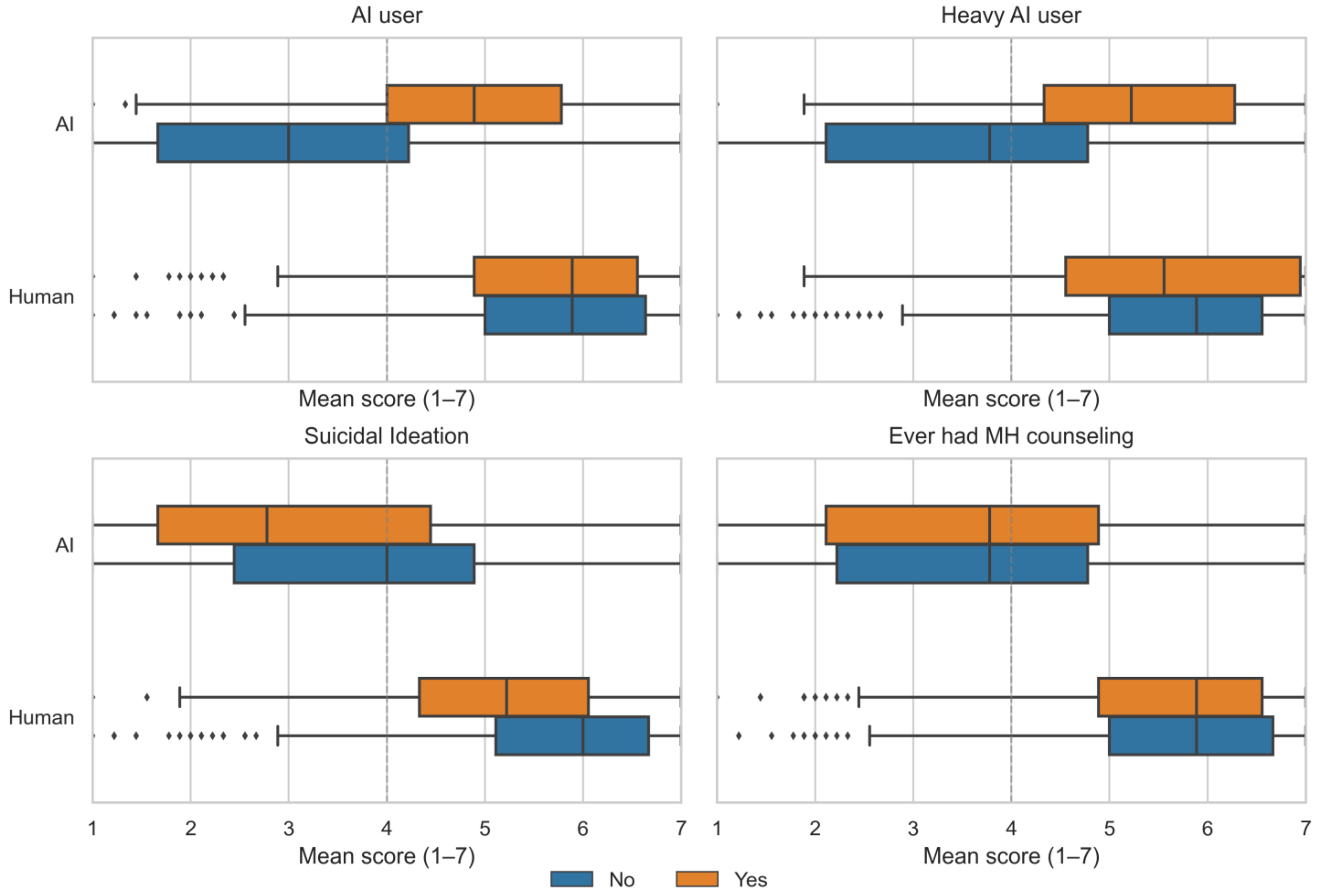

Notes: Each box represents the interquartile range (IQR; 25th–75th percentiles). The horizontal line inside the box indicates the median, and the whiskers extend to 1.5 × IQR. Circles represent individual data points outside that range. Higher scores indicate more favorable attitudes toward the specified source.
